# COVID-19 symptomatology and compliance with community mitigation strategies in Latin America early during the COVID-19 pandemic

**DOI:** 10.1016/j.pmedr.2021.101665

**Published:** 2021-12-10

**Authors:** Percy Herrera-Añazco, Diego Urrunaga-Pastor, Vicente A. Benites-Zapata, Guido Bendezu-Quispe, Carlos J. Toro-Huamanchumo, Adrian V. Hernandez

**Affiliations:** a Universidad Privada San Juan Bautista, Lima, Peru; b Instituto de Evaluación de Tecnologías en Salud e Investigación – IETSI, EsSalud, Lima, Peru; c Red Internacional en Salud Colectiva y Salud Intercultural, México, Mexico; d Universidad Científica del Sur, Lima, Peru; e Universidad San Ignacio de Loyola, Unidad para la Generación y Síntesis de Evidencias en Salud, Lima, Peru; f Universidad Privada Norbert Wiener, Centro de Investigación Epidemiológica en Salud Global, Lima, Peru; g Universidad Peruana de Ciencias Aplicadas, Lima, Peru; h Clínica Avendaño, Unidad de Investigación Multidisciplinaria, Lima, Peru; i University of Connecticut/Hartford Hospital Evidence-based Practice Center, Hartford, CT, USA; j Universidad San Ignacio de Loyola, Unidad de Revisiones Sistemáticas y Metaanálisis, Guías de Práctica Clínica y Evaluaciones Tecnológicas Sanitarias, Lima, Peru

**Keywords:** COVID-19, SARS-CoV-2, Social networking, Hand disinfection, Masks, Physical distancing, Latin America, Pandemics, Coronavirus infections

## Abstract

**Introduction:**

Community mitigation strategies (CMS) have demonstrated to be effective in the reduction of transmission and incidence of COVID-19, especially in the population with symptoms associated with the disease. The aim of this study was to evaluate the association between the presence of COVID-19 symptoms and adherence to CMS in Latin American adults.

**Methods:**

We carried out a secondary analysis of a database developed by the University of Maryland and Facebook social network during the COVID-19 pandemic. We included Latin American adults that used the Facebook platform and participated in a survey conducted from April 23 to May 23, 2020. The principal outcome variable was reported compliance with the three main CMS (physical distancing, use of face masks, and hand washing). The exposure variable included symptoms suspicious for COVID-19 defined as the presence of three or more symptoms of an acute clinical case of COVID-19. We performed generalized linear models of the Poisson family with a logarithmic link function to evaluate the association between the presence of COVID-19 symptoms and reported compliance with CMS. We calculated crude and adjusted prevalence ratios (PR) with their 95% confidence intervals (95%CI).

**Results:**

We analyzed 1,310,690 adults from Latin America; 48.1% were male and 42.9% were under 35 years of age. The prevalence of suspicious symptoms of COVID-19 was 18.5% and reported compliance with the three CMS was 45.3%. The countries with the highest proportion of reported compliance with the three CMS were Peru, Bolivia and Panama, while those with the lowest reported compliance were Costa Rica, Nicaragua and Honduras. In the adjusted model, people with suspicious symptoms for COVID-19 had a 14% lower compliance with the three CMS (aPR = 0.86; 95%CI: 0.85–0.87; p < 0.001).

**Conclusions:**

Less than half of the participants complied with the CMS, and those presenting suspicious symptoms for COVID-19 had lower reported compliance with the three CMS.

## Highlights

• Community mitigation strategies (CMS) constitute an effective tool to reduce the spread of infection and avoid the oversaturation of health services.
• The prevalence of suspicious symptoms of COVID-19 was 18.5%, and compliance with the three CMS was 45.3%.
• The countries with the highest proportion of compliance with the three CMS were Peru, Bolivia and Panama, while those with the lowest compliance were Costa Rica, Nicaragua and Honduras.
• People with suspicious symptoms of COVID-19 had a 14% lower compliance with CMS.

## 1 Introduction

To date, the COVID-19 pandemic covers 235 countries, and more than 64 million cases have been registered with more than 1.5 million deaths (World Health Organization, 2021). This crisis has implied significant changes in people's lifestyles, unleashing huge economic costs in nations (Nicola et al., 2020), both in high, low- and middle-income countries, where the population has been forced to continue their usual lifestyles to maintain their incomes. Likewise, the precarious health systems with limited human and economic resources (Castro, 2020, Americas Society/Council of the Americas, 2021) in these countries have been collapsed due to the great demand for hospitalizations due to COVID-19, with both factors inducing an increase in the death toll (World Health Organization, 2021).

Despite efforts to develop drugs for the treatment of COVID-19, to date, only supportive treatment is available (Esposito et al., 2020). In addition, the distribution of the vaccines now available requires effective logistic support which is greatly lacking in low-income countries (Frederiksen et al., 2020). In the meantime, community mitigation strategies (CMS) constitute an effective tool to reduce the spread of infection and avoid the oversaturation of health services, with these strategies being adopted and prioritized as public policy to reduce the contagion curve worldwide (Ngonghala et al., 2019).

CMS include social distancing, the use of masks, eye protection, and hand washing, which have been shown to be effective in reducing the incidence of COVID-19 (Chu et al., 2020). A systematic review of 172 observational studies in 16 countries showed that viral transmission was lower among those who maintained a distance of more than one meter compared to those who maintained a shorter distance. Likewise, transmission was lower among those who wore a mask and those who wore eye protection (Chu et al., 2020). However, the effectiveness of some of these measures varies according to the socio-economic and cultural context of nations. A study that included 149 countries found that although social distancing decreased the incidence of infection by 13%, social distancing was higher in countries with a higher gross domestic product, a greater number of patients over 65 years of age, and a higher health safety index (Islam et al., 2020).

In addition to these factors, another factor described as influencing the effectiveness of CMS is adherence to these measures, which also varies according to the study context. A study carried out in the African-American population in the United States found that 72% of those evaluated always washed their hands, 67% always maintained social distancing and 65% always wore a mask in public, suggesting a certain ethnic component in adherence (Block et al., 2020). Other studies also showed ethnic variations in adherence to CME. In a study carried out during Thanksgiving and winter break, Non-Hispanic Whites were more likely to gather with non-household members than were Hispanics or non-Hispanic Blacks. Mask wearing was more common among older participants, non-Hispanic Blacks, and Hispanics when gatherings included non-household members. Likewise, they observed high rates of face mask using in April. Then, in May, this increased further among non-Hispanic black, Hispanic or Latinos, and non-Hispanic of another race (Peacock et al., 2021). Similarly, adherence to different CMS also differs, with the use of a mask and hand washing being the most frequent practices as shown by some studies in Brazil and China (Tong et al., 2020, Lima-Costa et al., 2020, Lennon et al., 2020). It has also been suggested that sociocultural aspects, such as generational differences, educational level or where the information is obtained about disease may influence persistent adherence to CMS (Ahmed et al., 2020, Masters et al., 2020). It is very important to identify the factors associated with CMS adherence, then, the messages can be more effective.

Adherence to CMS becomes especially important in subjects with suspected clinical symptoms of COVID-19, defined according to the World Health Organization (WHO) as the presence of three or more symptoms including fever, cough, general weakness/fatigue, headache, myalgia, sore throat, coryza, dyspnea, anorexia/nausea/vomiting, diarrhea, and altered mental status (World Health Organization, 2020). Latin America is a heterogeneous region with respect to socio-cultural and economic aspects, with limitations in health structure, governance problems and qualified human resources that could influence adherence to CMS among people with clinical suspicion of COVID-19 (Castro, 2020, Americas Society/Council of the Americas, 2021). Given that adherence to CMS could be a potentially cost-effective strategy to reduce virus transmission (Burki, 2020, Andrus et al., 2020), the objective of this research was to evaluate the association between the presence of COVID-19 symptoms and adherence to CMS to avoid the transmission of the COVID-19 in Latin America.

## 2 Methods

### 2.1 Study design

We conducted a secondary data analysis of a database generated by the University of Maryland in conjunction with the social network Facebook (Facebook, Inc) through a survey conducted to obtain population information on different aspects in the context of the COVID-19 pandemic. This survey consists of five modules that include sociodemographic information, contact report, general health, mental health and economic security. The survey was conducted for the first time on April 23, 2020 and is translated into the languages and adaptations of the regions that use Facebook (Barkay et al., 2020).

### 2.2 Population and sample

The population studied included people 18 years of age or over who used the Facebook platform. Only the population of Latin America was included, which corresponded to 20 countries and 1,440,586 Facebook users. The people who answered the questionnaire of the health module and contact report were considered for the analysis, and those who did not fulfill the variables of interest were excluded. The effective sample analyzed was 1,310,690 adults from Latin America. The analysis period comprised the surveys from April 23 to May 23, 2020. The survey was translated into the predominant language in each country and region in which it was applied. Participants were selected randomly according to the sampling frame that Facebook estimated each day. If a Facebook user declined to fill the survey, another participant within the sampling frame was randomly selected and invited. Facebook users can only answer the survey once each eight weeks (Barkay et al., 2020). In addition, we added the survey in supplementary material.

The details of the weighting methodology have been described by Barkay et al (Barkay et al., 2020). Briefly, it is based on a two-stage weighting process. In the first stage, inverse propensity score weighting was employed to correct for non-response bias by increasing the sample's representativeness of the Facebook user sampling frame. In the second stage, poststratification or raking was used to compare the distribution of age and gender among Facebook users to UN Population Division (2019) World Population Projections benchmarks and first administrative level region benchmarks using publicly available population density maps.

### 2.3 Variables and procedure

#### 2.3.1 Primary outcomes

Physical distancing: Compliance was considered when participants reported “not having been in direct contact (including touching, shaking hands, hugging, kissing) for more than 1 min and not having been within 2 m of any person with whom you are not currently living in the last 24 h”. The survey question was “In the last 24 h, have you had direct contact with anyone who is not staying with you?”

Hand washing: Compliance was evaluated with the survey question “In the last 7 days, how often did you wash your hands with soap after being in public?” and the possible answers were: all of the time, most of the time, about half of the time, some of the time, none of the time and I have not been in public during the last 7 days. Compliance with hand washing was defined as whether the participant answered any of the first four responses.

Use of a mask: Compliance with mask using was evaluated with the survey question: “In the last 7 days, how often did you wear a mask when in public?” and the possible responses were: all of the time, most of the time, about half of the time, some of the time, none of the time and I have not been in public during the last 7 days. Compliance with mask using was defined as whether the participant answered any of the first four responses.

Likewise, an outcome composed of the fulfillment of the three primary outcomes was considered.

#### 2.3.2 Secondary outcomes

Quarantine due to exposure to respiratory symptomatic contact: Compliance was considered when the participants reported having remained isolated at home after having been in contact with someone with respiratory symptoms compatible with an acute symptomatic case of COVID-19 in the last 7 days.

Quarantine due to respiratory symptoms: Compliance was considered when the participants reported having remained isolated at home after having presented respiratory symptoms compatible with an acute symptomatic case of COVID-19 in the last 24 h.

#### 2.3.3 Exposure

Suspicious symptoms of COVID-19: This was defined when the participants reported the presence of three or more symptoms compatible with acute COVID-19 disease (fever, cough, shortness of breath, fatigue, coryza, muscle pain, sore throat, chest pain, nausea, loss of smell, eye pain, and headache) according to the WHO definition of a suspected case (World Health Organization, 2020).

#### 2.3.4 Other covariates

We analyzed gender (male, female, others), age group (18–24, 25–34, 35–44, 45–54, 55–64, 65–74, 75 or more years), and area of residence (city, town, rural area). Likewise, we evaluated the presence of depressive symptoms (survey question: How often did you feel so depressed that nothing could cheer you up in the past seven days?) and anxiety symptoms (survey question: During the last seven days, how often did you feel so nervous that nothing could calm you down?) These questions had five response alternatives: all the time, most of the time, some of the time, a little of the time, and none of the time. Then, we considered the first four alternatives as depressive or anxiety symptomatology, respectively. This two questions were adapted from the Kessler Psychological Distress Scale (*K*10) and evaluated the anxiety/nervous and depressive symptoms within a period of seven days (Andrews and Slade, 2001). We considered these two variables of particular importance because previous studies have shown a relationship between both depression and anxiety with the fulfillment of some mitigation strategies or risk behaviors during the COVID-19 pandemic (Ebrahimi et al., 2021, Wang et al., 2020, Shiina et al., 2020).

In addition, we included the level of CMS applied at each country when the survey was performed. We considered three possible categories: low (defined when they only applied partial measures as closing educational centers and social distancing recommendations), intermediate (defined when they applied a lockdown in only some areas) and high (defined when the country was under complete quarantine) (Bolaño-Ortiz et al., 2020).

### 2.4 Statistical analysis

The database was downloaded in Microsoft Excel 2010 format files and imported into the statistical package STATA v14.0 (StataCorp, TX, USA). All analyses were carried out considering the complex sampling of the survey using the svy command of the statistical software.

The qualitative variables were described using absolute frequencies and weighted proportions according to complex sampling with their 95% confidence intervals (95%CI). We performed bivariate analysis between the covariates and the main variable of exposure or outcomes, using the Pearson's Chi square test with Rao-Scott correction, considering the complex sampling of the survey. We performed generalized linear models of the Poisson family with a logarithmic link function to evaluate the association between the outcomes (primary and secondary) and COVID-19 symptoms. Crude and adjusted prevalence ratios (PR) were calculated with their 95%CIs, and an epidemiological approach (confounders were defined as variables associated with the outcome and the exposure according to previous studies and not in the causal path) was considered for entering the variables of the adjusted model. In addition, due to the possibility of selection bias, we performed a sensitivity analysis between the participants who had missing and no missing data according to the variables of interest (supplementary material).

### 2.5 Ethical aspects

The database was downloaded without identifiers by one of the researchers, ensuring the privacy of the participants was not compromised.

## 3 Results

### 3.1 Characteristics of the study population

We analyzed the data of 1,310,690 adults from Latin America from April to May 2020. The total number of participants by country is presented in Supplementary Table 1. Of these, 1,310,690 adults, 48.1% (n = 580,426) were male, and 42.9% (n = 715,155) were under 35 years of age. The proportion of participants with anxiety and depressive symptoms was 44.7% (n = 625,860) and 46.6% (n = 663,934), respectively, and the prevalence of suspicious symptoms of COVID-19 was 18.5% (n = 274,306). Significant differences were found among the covariates included in the analysis according to the presence of COVID-19 symptoms as shown in Table 1.

**Table 1:** Descriptive and bivariate analysis of the study sample characteristics according to COVID-19 symptoms (n = 1,310,690; N = 11,267,524).

				**COVID-19 symptomatology**				
	Total			Yes		No		
Characteristics	Absolute frequency of participants surveyed	Weighted proportion according to each category		Weighted proportion according to each category		Weighted proportion according to each category		p value
	N	%	95%CI	%	95%CI	%	95%CI	
Gender								<0.001
Male	580,426	48.1	47.7–48.5	40.2	39.4–41.0	49.9	49.5–50.3	
Female	715,989	50.5	50.1–50.9	58.4	57.6–59.2	48.7	48.3–49.1	
No binary	14,275	1.4	1.2–1.7	1.4	1.2–1.6	1.4	1.2–1.7	
Age (years)								<0.001
18–24	310,465	18.1	17.4–18.8	27.2	26.3–28.3	16.0	15.3–16.7	
25–34	404,690	24.8	24.1–25.5	30.0	29.4–30.7	23.6	22.9–24.3	
35–44	277,273	18.7	18.4–19.0	18.3	17.7–18.8	18.8	18.5–19.1	
45–54	175,466	18.7	18.4–19.0	14.6	14.0–15.2	19.7	19.3–20.0	
55–64	102,144	11.1	10.7–11.5	6.1	5.7–6.5	12.3	11.8–12.7	
65–74	34,735	7.4	6.9–8.0	3.2	2.9–3.6	8.4	7.8–9.1	
75 years or older	5,917	1.2	1.1–1.3	0.5	0.4–0.7	1.3	1.2–1.5	
Area of residence								<0.001
City	1,030,744	78.9	75.7–81.8	81.8	78.6–84.6	78.3	75.1–81.1	
Town	182,088	13.8	11.5–16.5	12.2	9.9–15.0	14.2	11.9–16.9	
Village or rural area	97,858	7.3	6.6–8.0	6.0	5.4–6.6	7.5	6.8–8.3	
Anxiety symptomatology								<0.001
Yes	625,860	44.7	44.0–45.3	65.6	65.0–66.3	39.9	39.3–40.5	
No	684,830	55.3	54.7–56.0	34.4	33.7–35.1	60.1	59.5–60.7	
Depressive symptomatology								<0.001
Yes	663,934	46.6	45.9–47.4	68.7	68.0–69.5	41.6	40.8–42.4	
No	646,756	53.4	52.6–54.1	31.3	30.5–32.1	58.4	57.6–59.2	
Level of CMS applied								<0.001
Low	315,823	20.1	11.9–32.0	20.7	12.1–33.1	20.0	11.9–31.8	
Intermediate	463,658	41.0	27.9–55.5	45.1	30.9–60.1	40.0	27.2–54.4	
High	531,209	38.9	28.5–50.4	34.2	24.3–45.7	40.0	29.5–51.4	

95%CI: 95% confidence intervals.

### 3.2 Prevalence of CMS compliance

The prevalence of reported compliance with the three CMS as a whole was 45.3% (n = 582,210), and significant differences were found with respect to the covariates of interest, except for anxiety symptoms (p = 0.275). Likewise, 38.9% (n = 106,556) of the participants presented suspicious symptoms for COVID-19 and complied with the three CMS (Table 2).

**Table 2:** Bivariate analysis of the sample characteristics according to compliance with the principal community mitigation strategies in the study sample.

	**Compliance with the principal mitigation measures**				
	Yes		No		
Characteristics	Weighted proportion according to each category		Weighted proportion according to each category		p value
	%	95%CI	%	95%CI	
Gender					<0.001
Male	44.6	43.6–45.7	55.4	54.3–56.4	
Female	45.9	44.7–47.1	54.1	52.9–55.3	
No binary	43.5	40.7–46.3	56.5	53.7–59.3	
Age (years)					<0.001
18–24	37.4	36.0–38.9	62.6	61.1–64.0	
25–34	42.8	41.3–44.4	57.2	55.6–58.7	
35–44	47.2	45.7–48.7	52.8	51.3–54.3	
45–54	50.9	50.1–51.7	49.1	48.3–49.9	
55–64	50.4	49.4–51.4	49.6	48.6–50.6	
65–74	46.7	45.3–48.0	53.3	52.0–54.7	
75 years or older	39.0	37.1–41.0	61.0	59.0–62.9	
Area of residence					<0.001
City	46.1	44.8–47.3	53.9	52.7–55.2	
Town	42.6	41.5–43.8	57.4	56.2–58.5	
Village or rural area	41.4	40.1–42.8	58.6	57.2–60.0	
Anxiety symptomatology					0.275
Yes	45.0	43.5–46.5	55.0	53.5–56.5	
No	45.5	44.6–46.4	54.5	53.6–55.4	
Depressive symptomatology					<0.001
Yes	44.0	42.4–45.5	56.0	54.5–57.6	
No	46.4	45.6–47.2	53.6	52.8–54.4	
COVID-19 symptomatology					<0.001
No	46.7	45.7–47.7	53.3	52.3–54.3	
Yes	38.9	37.5–40.4	61.1	59.6–62.5	
Level of CMS applied					0.002
Low	43.1	42.2–44.1	56.9	55.9–57.8	
Intermediate	44.4	42.5–46.4	55.6	53.6–57.5	
High	47.3	46.1–48.5	52.7	51.5–53.9	

95%CI: 95% confidence intervals.

When considering the three CMS of hand washing, use of masks and physical distancing separately, compliance among the participants was 86.6% (n = 1,149,445), 82.9% (n = 1,098,057) and 59.6% (n = 754,338), respectively (Table 3), while among participants with suspicious symptoms for COVID-19 compliance with these strategies was 89.6% (n = 246,353), 85.3% (n = 234,990) and 49.3% (n = 134,727), respectively. It was found that physical distancing was more frequently carried out by females and those of non-binary gender. However, male participants more frequently complied with hand washing and use of a mask. Table 3 shows the statistically significant differences among the proportions of the covariates evaluated according to the main outcomes.

**Table 3:** Bivariate analysis of the study sample characteristics according to different community mitigation strategies (n = 1,310,690; N = 11,267,524).

	**Physical distancing**					**Hand washing**					**Mask or face covering use**				
	Yes		No			*Yes*		No			Yes		*No*		
Characteristics	Weighted proportion according to each category		Weighted proportion according to each category		p value	Weighted proportion according to each category		Weighted proportion according to each category		p value	Weighted proportion according to each category		Weighted proportion according to each category		p value
	%	95%CI	%	95%CI		%	95%CI	%	95%CI		%	95%CI	%	95%CI	
Gender					<0.001					<0.001					<0.001
Male	55.7	54.4–57.0	44.3	43.0–45.6		89.9	88.9–90.9	10.1	9.1–11.1		86.2	84.4–87.7	13.8	12.3–15.6	
Female	63.1	61.6–64.7	36.9	35.3–38.4		83.6	82.4–84.8	16.4	15.2–17.6		80.1	78.5–81.6	19.9	18.4–21.5	
No binary	66.7	63.1–70.0	33.3	30.0–36.9		78.4	75.8–80.7	21.6	19.3–24.2		72.8	69.2–76.1	27.2	23.9–30.8	
Age (years)					<0.001					<0.001					<0.001
18–24	53.5	51.7–55.3	46.5	44.7–48.3		84.5	83.3–85.5	15.5	14.5–16.7		78.4	76.3–80.4	21.6	19.6–23.7	
25–34	54.1	52.5–55.7	45.9	44.3–47.5		89.7	88.9–90.4	10.3	9.6–11.1		85.6	83.6–87.4	14.4	12.6–16.4	
35–44	58	56.1–59.9	42	40.1–43.9		90.2	89.2–91.1	9.8	8.9–10.8		87.2	85.5–88.7	12.8	11.3–14.5	
45–54	63.4	62.0–64.8	36.6	35.2–38.0		88.5	87.1–89.7	11.5	10.3–12.9		86.0	84.5–87.4	14.0	12.6–15.5	
55–64	67.8	66.5–69.1	32.2	30.9–33.5		83.6	81.8–85.4	16.4	14.6–18.2		81.4	79.7–82.9	18.6	17.1–20.3	
65–74	72.9	71.8–74.0	27.1	26.0–28.2		75.1	73.3–76.8	24.9	23.2–26.7		72.1	70.1–74.0	27.9	26.0–29.9	
75 years or older	73.8	71.3–76.1	26.2	23.9–28.7		65.5	62.9–67.9	34.5	32.1–37.1		60.2	58.0–62.4	39.8	37.6–42.0	
Area of residence					<0.001					<0.001					<0.001
City	59.3	57.7–60.9	40.7	39.1–42.3		87.6	86.5–88.6	12.4	11.4–13.5		84.3	82.5–86.0	15.7	14.0–17.5	
Town	59.4	58.3–60.5	40.6	39.5–41.7		84.3	83.7–84.9	15.7	15.1–16.3		79.5	77.7–81.2	20.5	18.8–22.3	
Village or rural area	63.4	62.0–64.8	36.6	35.2–38.0		79.9	78.8–81.0	20.1	19.0–21.2		73.7	71.4–75.9	26.3	24.1–28.6	
Anxiety symptomatology					<0.001					<0.001					<0.001
Yes	57.7	55.9–59.4	42.3	40.6–44.1		88	87.0–88.9	12	11.1–13.0		84.6	82.9–86.1	15.4	13.9–17.1	
No	61.1	60.0–62.4	38.8	37.6–40.0		85.4	84.2–86.6	14.6	13.4–15.8		81.6	79.8–83.2	18.4	16.8–20.2	
Depressive symptomatology				<0.001					<0.001					<0.001	
Yes	57.2	55.5–58.9	42.8	41.1–44.5		87.4	86.4–88.3	12.6	11.7–13.5		83.7	82.0–85.4	16.3	14.6–18.0	
No	61.7	60.5–62.9	38.3	37.1–39.5		85.9	84.6–87.1	14.1	12.9–15.4		82.2	80.5–83.8	17.8	16.2–19.5	
COVID-19 symptomatology				<0.001					<0.001					<0.001	
No	62.0	60.6–63.3	38.0	36.7–39.4		85.9	84.7–87.0	14.1	13.0–15.3		82.4	80.7–83.9	17.6	16.1–19.3	
Yes	49.3	47.8–50.8	50.7	49.2–52.2		89.6	88.8–90.4	10.4	9.6–11.2		85.3	83.1–87.2	14.7	12.8–16.9	
Level of CMS applied					<0.001					<0.001					<0.001
Low	58.5	56.0–60.9	41.5	39.1–44.0		86.5	86.0–87.0	13.5	13.0–14.0		80.7	78.1–83.1	19.3	16.9–21.9	
Intermediate	55.5	54.2–56.7	44.5	43.3–45.8		90.8	90.2–91.4	9.2	8.6–9.8		85.4	80.9–89.0	14.6	11.0–19.1	
High	64.6	63.3–65.9	35.4	34.1–36.7		82.2	81.5–82.9	17.8	17.1–18.5		81.4	80.4–82.3	18.6	17.7–19.6	

95%CI: 95% confidence intervals.

Regarding secondary outcomes, the proportion of participants who completed quarantine due to contact with a patient with COVID-19 was 16.1% (n = 10,686) and 18.8% (n = 55,072) for those who completed quarantine for being symptomatic. Likewise, only 16.7% (n = 5,955) of the participants who presented symptoms of COVID-19 remained in quarantine after being exposed to a contact with COVID-19. Significant differences were found among gender (p < 0.001), age (p < 0.001), depressive symptomatology (p = 0.001) and COVID-19 symptomatology (p = 0.036) regarding the status of quarantine due to contact with a respiratory symptomatic. Similarly, significant differences among gender (p < 0.001), age (p = 0.010) and area of residence (p = 0.009) were found for quarantine due to symptoms (Table S3).

### 3.3 Prevalence of CMS compliance according to countries

**Fig. 1:**
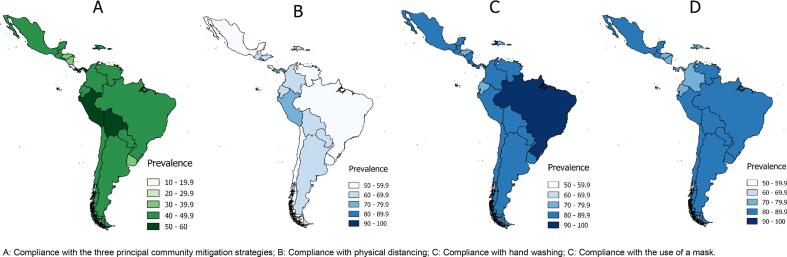
Prevalence of compliance with community mitigation strategies according to each country in Latin America. A: Compliance with the three principal community mitigation strategies; B: Compliance with physical distancing; C: Compliance with hand washing; C: Compliance with the use of a mask.

The countries with the highest proportion of reported compliance with the three CMS were Peru (54.1%), Bolivia (52.1%), Panama (51.9%), Puerto Rico (51.2%) and Argentina (49.5%). On the other hand, those with the lowest reported compliance were Costa Rica (17.3%), Nicaragua (31.4%), Honduras (38.8%), Uruguay (39.3%) and Haiti (41.3%) (Fig. 1 and Table S1).

**Fig. 2:**
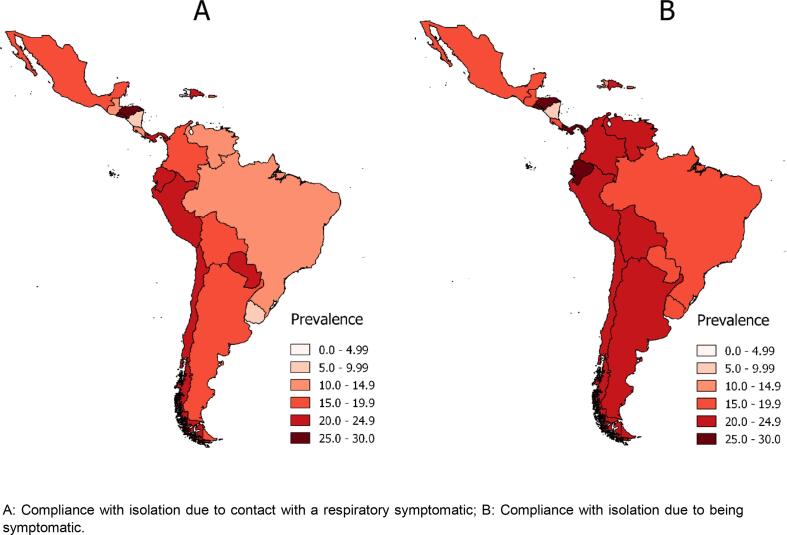
Prevalence of compliance with secondary outcomes according to each country in Latin America. A: Compliance with isolation due to contact with a respiratory symptomatic; B: Compliance with isolation due to being symptomatic.

Regarding compliance with quarantine due to having been in contact with a suspected case, the countries with the best compliance were Honduras (28.2%), El Salvador (25.3%), Ecuador (23.4%), Chile (23.2%) and Panama (22.7 %) while those with the lowest compliance were Haiti (1.5%), Uruguay (8.4%), Nicaragua (9.3%), Costa Rica (10.7%) and Venezuela (13.0%). The countries showing better compliance with quarantine due to being symptomatic were Panama (29.0%), Ecuador (26.9%), Honduras (26.7%), El Salvador (25.9%) and the Dominican Republic (24.2%), with Nicaragua (10.0%), Haiti (10.9%), Uruguay (15.2%), Costa Rica (15.2%) and Guatemala (16.4%) presenting the worst compliance (Fig. 2 and Table S2).

### 3.4 Association between suspicious symptoms of COVID-19 and compliance with CMS

In the adjusted model, in people with suspicious symptoms of COVID-19, compliance of the three CMS was 15% less likely in those with symptoms of COVID-19 compared to those who did not present symptoms (aPR = 0.85; 95%CI: 0.84–0.87; p < 0.001). Similarly, when analyzing compliance with CMS separately, it was found that physical distancing was 18% less likely in those with suspicious symptoms for COVID-19 (aPR = 0.82; 95%CI: 0.81–0.83; p < 0.001). On the contrary, they had a greater probability of complying with hand washing (aPR = 1.03; 95%CI: 1.03–1.04; p < 0.001) and the use of a mask (aPR = 1.03; 95%CI: 1.02–1.03; p < 0.001) (Table 4).

**Table 4:** Generalized linear models of the Poisson family to evaluate the association between COVID-19 symptoms and compliance with community mitigation strategies in the study sample.

***Primary outcome: Compliance with the principal mitigation measures***	Crude			Adjusted		
COVID-19 symptomatology	cPR	95%CI	p value	aPR*	95%CI	p value
No	Reference	–	–	Reference	–	–
Yes	0.83	0.82–0.85	<0.001	0.86	0.85–0.87	<0.001
***Primary outcome: Physical distancing***						
COVID-19 symptomatology						
No	Reference	–	–	Reference	–	–
Yes	0.80	0.78–0.81	<0.001	0.83	0.82–0.84	<0.001
***Primary outcome: Hand washing***						
COVID-19 symptomatology						
No	Reference	–	–	Reference	–	–
Yes	1.04	1.04–1.05	<0.001	1.03	1.02–1.04	<0.001
***Primary outcome: Mask or face covering use***						
COVID-19 symptomatology						
No	Reference	–	–	Reference	–	–
Yes	1.04	1.03–1.05	<0.001	1.03	1.02–1.03	<0.001
***Secondary outcome: Isolation due to contact with a respiratory symptomatic***						
COVID-19 symptomatology						
No	Reference	–	–	Reference	–	–
Yes	1.08	1.01–1.17	0.035	1.05	0.98–1.14	0.186

95%CI: 95% confidence intervals; cPR: Crude prevalence ratio; aPR: Adjusted prevalence ratio.

*Adjusted for: gender, age, area of residence, anxiety, depression symptomatology and level of CMS applied.

## 4 Discussion

Our findings show that only four out of every ten persons with suspicious symptoms for COVID-19 complied with the three CMS evaluated in the study (physical distancing, hands washing, and use of face mask). Differences in reported compliance with CMS were also identified among the Latin American countries included in the study. The three countries presenting the highest prevalence of reported compliance with the CMS evaluated were Panama, Peru, and Bolivia, while Costa Rica, Nicaragua, and Uruguay showed the lowest prevalence of compliance.

America is one the regions most affected by the COVID-19 pandemic, with the greatest number of infected people worldwide up to December 12 and a total of 29 million confirmed cases compared to Europe with 21 million cases of infected patients (World Health Organization, 2021). Similarly, up to May 25, the end date of the study period for this research and almost one month after the beginning of the quarantine in the majority of Latin American countries, there were more than 2.7 million confirmed cases compared to Europe in which 2.1 million cases had been reported (World Health Organization, 2021). The temporary framework of our study is placed at the beginning of the obligatory measures for social distancing ordered by Latin American governments. This could explain why less than a half of the participants with suspicious symptoms for COVID-19 complied with the three CMS and only half of these individuals maintained physical distancing. Although the reasons why social distancing was the less adherent CMS in LAC have not been studied in our research, other studies can help us understand our findings. A previous study carried out in North America and Europe found that the most frequently motivations against social distancing (or barriers) included “There are many people walking on the streets in my area”, “I have friends or family who need me to run errands for them”, “I don't trust the messages my government provides me about the pandemic”, and “I feel stressed when I am alone or isolated” (Coroiu et al., 2020). Specifically in Latin American countries, a previous research found risk perceptions of COVID-19 were related to household income, COVID-19 incidence, perceived preparedness of the health care system. However, risk perceptions do not seem to influence the decision to stay at home (Alicea-Planas et al., 2021).

The variation in compliance with CMS may be related to the measures established by each country to mitigate the spread of the virus, their effect and the social determinants of health in each country (Benítez et al., 2020, Martinez-Valle, 2021). Although the response of all LAC countries was not analyzed, previous studies showed how was the response of the governments in Argentina, Brazil, Chile, Ecuador, Colombia, Mexico and Peru. Overall, the studies showed that these countries rushed to implement strict control measures against COVID-19 and gradually increased the capacity of their health systems (Benítez et al., 2020, Martinez-Valle, 2021). However, pre-pandemic conditions in their health systems, as well as socioeconomic indicators such as high unemployment and social inequalities, undermined the effectiveness of responses. Likewise, there was no comprehensive strategy for testing, monitoring and tracing cases, which contributed to do not contain adequately the spread of the virus (Benítez et al., 2020, Martinez-Valle, 2021). Similarly, economic support measures were late implemented and were too timid for most countries. Then, this five countries experienced a large number of cases and deaths, which in some cases were much higher than the official reports (Benítez et al., 2020, Martinez-Valle, 2021).

Despite experience with previous epidemics caused by other viruses, this new coronavirus raised concerns with initially divergent responses with regard to the transmission of the virus, its presence on surfaces or the need for the use of face masks, thereby generating unclear messages about CMS in initial information campaigns led by different governments and even with involuntary communication errors by the WHO (Alvarez-Risco et al., 2020, Pascarella et al., 2020, Riggioni et al., 2020, Soave, 2020).

Accordingly, the WHO implemented a checklist for the preparation of communication and community participation for response to COVID-19 in different countries (Ebrahimi et al., 2021) Communication should be an effective and accurate, with punctual information spreading for those people at risk (Alvarez-Risco et al., 2020). However, without clear initial concepts, the information initially provided likely contributed to the confusion that limited effective follow-up to the recommendations. A British study criticized governmental communication, expressing a lack of confidence in the government and a lack of clarity around social distancing and quarantine guidelines (Fridman et al., 2020). However, assessment of the response to these communication strategies by following social networks could lead to improvements over time which could vary from country to country as recently demonstrated by a comparative study of communication strategies that circulated in Facebook during the pandemic and was related to government mechanisms in the United States, Singapore and England (Heine et al., 2002). To our knowledge, there are no reports in Latin America on the assessment of population responses to CMS disseminated through traditional and digital communication media.

Although the messages should be accurate and punctual, their individualization with regard to aspects such as the age of the target groups is also very important. Our study identified that older participants more closely complied with measures of physical distancing while presenting less compliance with hand washing and the use of face masks. The recommendations against COVID-19 were widely spread through social networks, but these media are less frequently used by older adults, and they are not necessarily trusted by this age group (Fridman et al., 2020). On the other hand, massive communication strategies do not take into account frequent conditions often found among older adults such as frailty, health literacy, hearing loss, eye problems or dementia, and thereby limit their effectiveness (Heine et al., 2002, van Vliet et al., 2015). Our results are similar to those presented by a North American study that identified that despite older adults having a lower perception of risk than young people, they presented higher levels of compliance with physical distancing (Masters et al., 2020).

The need to implement public health policies that are sensitive to gender has been recognized, with communication and promotion strategies specific to this group. Regular communication can reinforce stereotypes and not necessarily focus on women or people of non-binary gender, which limits the effectiveness of communication strategies. (Oertelt-Prigione et al., 2017, Hasan and Gil, 2016). During the quarantine period in Peru, a strategy known as “peak and gender” was implemented that allowed only men and women to go outside on specific days. This strategy led to agglomerations in the markets on the days women were allowed to go out, since in this country and probably in many Latin American countries, women are traditionally in charge of domestic purchases, exposing them to infections because physical distancing is not maintained (Reisman, 2020). Other studies have described that younger men were at a higher risk of refusing to adhere to government action. This finding can be explained due to lower levels of risk perception and higher levels of personality trait sensation seeking and lower risk perception could explain this finding (Margraf et al., 2020).

Similarly, a communication strategy that does not consider cultural, gender, generational, and even idiomatic differences cannot achieve the expected results. In New York, a communication strategy that included several communication media was implemented during the pandemic. In this strategy, a member of the advisory committee on health and the mayor were the main spokespersons. However, it was only relatively effective because the diversity of people living in that city with different languages and origins was not considered, limiting effective information spreading (Ataguba and Ataguba, 2020).

As described in studies conducted in Brazil and China, hand washing and the use of face masks were the CMS most commonly used by both the general population and people with symptoms suspicious for COVID-19 (Tong et al., 2020, Lima-Costa et al., 2020). However, although there are differences in reported compliance with CMS between countries, there may also be differences between ethnic groups or regions within each country. In a study among African Americans in the United States physical distancing was more frequent than the use of a mask, unlike our study, in which physical distancing was the CMS with the least adherence, which may be related to socioeconomic and cultural differences (Block et al., 2020). In Latin American countries, most of the population depends on informal jobs (Basto‑Aguirre et al., 2020​) that require contact with other people, making recommendations such as hand washing, confinement and physical distancing difficult to follow due to lack of access to water and soap during the workday (Castro, 2020). Similarly, variations in both the beginning of the implementation of the CMS and differences in the communication strategies in each country as well as their social determinants may explain the differences in adherence to CMS and isolation (Americas Society/Council of the Americas, 2021). In fact, although not all the countries established the same measures and many countries share common characteristics, it is evident that the pandemic impact was not equally in all of them (Ortiz-Prado et al., 2020).

Although a variation in prevalence was found when CMS were evaluated separately, it was of note that, in general, there was a reduction in compliance with CMS in the population with symptoms suspicious for COVID-19. Although we did not evaluate the reasons for this finding, this reduction might be explained by psychological aspects even though it would be expected that in the presence of COVID-19 symptoms people should be more concerned about their safety. In recently diagnosed diabetic patients we observed a phase process similar to the mourning process with an initial refusal to accept the diagnosis, thereby limiting adherence to treatment (Rodríguez-Moctezuma et al., 2015, Isla et al., 2008). Some patients feel threatened by the requirements of treatment, control of the disease and the consequences to their quality of lives, and therefore, decide not to follow the recommendations (Rodríguez-Moctezuma et al., 2015, Isla et al., 2008). It is likely that on becoming aware of having a suspicious clinical presentation of an unknown and potentially mortal disease, some patients adopted a denial phase with the corresponding reduction in adherence to CMS.

Our study has some limitations. First, despite being a multinational study with a significant sample size, it is based on the users of a social network to which not all people have access. However, it is a social network with widespread use in Latin America; for example, four out of every five Latin American Net users have a Facebook profile. Second, the variables included in the study and their definitions are subordinated to the pre-established definition presented in the survey matrix. Third, it is a self-reporting survey, and therefore, there could be a social desirability bias, generating a lower prevalence of exposure or outcome. Fourth, we evaluated the probability of selection bias with a sensitivity analysis, and we found statistically significant differences between the included and excluded participants. Fifth, we cannot establish causal relationships among the variables evaluated. Finally, certain variables such as perceived risk of severe disease, education level and income level were not measured by the survey. Nonetheless, this is the first multinational study with a significant sample size carried out in Latin America.

To conclude, less than a half of the participants complied with all the CMS for COVID-19 transmission. The participants that presented suspicious symptoms for COVID-19 showed lower reported compliance with the three CMS (physical distancing, use of face masks and hands washing). The results of this study show the need to send messages to increase adherence to CMS in countries of Latin America.

Reliable information is vital for designing and implementing preventive measures and promoting health awareness in the fight against COVID-19. Our study describes the need to design flexible communication strategies considering age groups and gender. This will be more effective to communicate preventive strategies for the virus spread in people with COVID-19.

## 5 Funding sources

This research did not receive any specific grant from funding agencies in the public, commercial or not-for-profit sectors.

## Author contributions

PHA, DUP, VBZ, GBQ, CTH, DUP and AVH have participated in the conception of the article, the data collection, its writing and approval of the final version. In addition, DUP and VABZ performed the data analysis.

## Declaration of Competing Interest

The authors declare that they have no known competing financial interests or personal relationships that could have appeared to influence the work reported in this paper.
