# Supplementary material for: COVID-19 symptomatology and compliance with community mitigation strategies in Latin America early during the COVID-19 pandemic
Source: Prev Med Rep. 2021 Dec 10;25:101665. doi: 10.1016/j.pmedr.2021.101665 (PMC8662838; doi:10.1016/j.pmedr.2021.101665)
Supplement: Supplementary Data 1 [file mmc1.docx]

| Table S1. Proportion of compliance with the main outcomes according to the countries included in the sample. | | | | | | | | | | | | | | | | | | | |
| --- | --- | --- | --- | --- | --- | --- | --- | --- | --- | --- | --- | --- | --- | --- | --- | --- | --- | --- | --- |
|  |  |  |  | **Compliance with the principal mitigation measures** | | | | **Physical distancing** | | | | **Hand washing** | | | | **Mask use** | | | |
|  | Total | | | Yes | | No | | Yes | | No | | Yes | | No | | Yes | | No | |
| Countries | n | Weighted proportion | IC95% | % | IC95% | % | IC95% | % | IC95% | % | IC95% | % | IC95% | % | IC95% | % | IC95% | % | IC95% |
| Argentina | 118,692 | 8.1 | 3.9-16.1 | 49.5 | 46.9-52.1 | 50.5 | 47.9-53.1 | 65.4 | 62.1-68.5 | 34.6 | 31.5-37.9 | 83.7 | 82.8-84.5 | 16.3 | 15.5-17.2 | 83.3 | 81.6-84.8 | 16.7 | 15.2-18.4 |
| Bolivia | 26,656 | 1.4 | 0.6-3.1 | 52.1 | 49.7-54.4 | 47.9 | 45.6-50.3 | 69.7 | 66.5-72.6 | 30.3 | 27.4-33.5 | 83.3 | 82.4-84.2 | 16.7 | 15.8-17.6 | 81.7 | 80.7-82.6 | 18.3 | 17.4-19.3 |
| Brazil | 386,010 | 37.1 | 24.0-52.6 | 44.9 | 42.7-47.2 | 55.1 | 52.8-57.3 | 55 | 53.7-56.3 | 45 | 43.7-46.3 | 91.4 | 90.7-92.0 | 8.6 | 8.0-9.3 | 86.7 | 81.1-90.8 | 13.3 | 9.2-18.9 |
| Chile | 55,547 | 3.4 | 1.6-6.8 | 47 | 44.9-49.2 | 53 | 50.8-55.1 | 59.1 | 55.8-62.4 | 40.9 | 37.6-44.2 | 86.8 | 85.7-87.9 | 13.2 | 12.1-14.3 | 87.2 | 86.2-88.2 | 12.8 | 11.8-13.8 |
| Colombia | 95,948 | 8.1 | 4.4-14.4 | 44 | 42.6-45.4 | 56 | 54.6-57.4 | 62.9 | 60.7-65.1 | 37.1 | 34.9-39.3 | 80.6 | 79.5-81.7 | 19.4 | 18.3-20.5 | 78.8 | 77.1-80.4 | 21.2 | 19.6-22.9 |
| Costa Rica | 26,920 | 0.9 | 0.4-1.8 | 17.3 | 15.6-19.1 | 82.7 | 80.9-84.4 | 56 | 52.1-59.8 | 44 | 40.2-47.9 | 87.2 | 85.9-88.4 | 12.8 | 11.6-14.1 | 32.3 | 28.0-37.0 | 67.7 | 63.0-72.0 |
| Dominican Republic | 17,076 | 1.5 | 0.8-2.9 | 44.4 | 42.6-46.2 | 55.6 | 53.8-57.4 | 63.9 | 60.5-67.2 | 36.1 | 32.8-39.5 | 78.9 | 77.3-80.3 | 21.1 | 19.7-22.7 | 79.3 | 77.4-81.1 | 20.7 | 18.9-22.6 |
| Ecuador | 45,526 | 2.6 | 1.3-5.0 | 46.5 | 44.8-48.2 | 53.5 | 51.8-55.2 | 68.2 | 64.6-71.6 | 31.8 | 28.4-35.4 | 77.5 | 76.0-78.9 | 22.5 | 21.1-24.0 | 77.1 | 75.1-78.9 | 22.9 | 21.1-24.9 |
| El Salvador | 26,575 | 1 | 0.5-2.1 | 47.2 | 46.0-48.3 | 52.8 | 51.7-54.0 | 73 | 71.0-75.0 | 27 | 25.0-29.0 | 73.8 | 71.1-76.4 | 26.2 | 23.6-28.9 | 73.4 | 70.8-75.9 | 26.6 | 24.1-29.2 |
| Guatemala | 31,194 | 2.4 | 1.3-4.4 | 48 | 46.4-49.6 | 52 | 50.4-53.6 | 63.1 | 61.3-64.8 | 36.9 | 35.2-38.7 | 83.7 | 82.9-84.5 | 16.3 | 15.6-17.1 | 86.5 | 85.6-87.4 | 13.5 | 12.6-14.4 |
| Haiti | 721 | 0.5 | 0.1-1.5 | 41.3 | 38.0-44.7 | 58.7 | 55.3-62.0 | 59.1 | 55.6-62.5 | 40.9 | 37.5-44.4 | 90.1 | 87.5-92.1 | 9.9 | 7.9-12.5 | 74.4 | 72.0-76.7 | 25.6 | 23.3-28.0 |
| Honduras | 20,599 | 1.3 | 0.7-2.6 | 38.8 | 36.8-40.9 | 61.2 | 59.1-63.2 | 65 | 62.3-67.7 | 35 | 32.3-37.7 | 73.2 | 71.3-75.0 | 26.8 | 25.0-28.7 | 70.5 | 68.9-72.2 | 29.5 | 27.8-31.1 |
| Mexico | 292,882 | 19.2 | 11.1-31.2 | 43.7 | 43.0-44.4 | 56.3 | 55.6-57.0 | 59.1 | 56.8-61.3 | 40.9 | 38.7-43.2 | 86.3 | 85.8-86.8 | 13.7 | 13.2-14.2 | 81.1 | 78.3-83.7 | 18.8 | 16.3-21.7 |
| Nicaragua | 22,941 | 0.9 | 0.4-1.9 | 31.4 | 27.0-36.1 | 68.6 | 63.4-73.1 | 46 | 43.0-49.1 | 54 | 50.9-57.0 | 89.8 | 88.6-90.8 | 10.2 | 9.2-11.4 | 71.7 | 64.0-78.4 | 28.3 | 21.6-36.0 |
| Panama | 8,590 | 0.6 | 0.3-1.4 | 51.9 | 49.3-54.5 | 48.1 | 45.5-50.7 | 71.3 | 67.7-74.6 | 28.7 | 25.4-32.3 | 81.6 | 80.7-82.5 | 18.4 | 17.5-19.3 | 78.6 | 77.0-80.0 | 21.4 | 20.0-23.0 |
| Paraguay | 10,591 | 0.8 | 0.4-1.7 | 44.7 | 42.8-46.5 | 55.3 | 53.5-57.2 | 61.2 | 57.6-64.7 | 38.8 | 35.3-42.4 | 83.1 | 81.5-84.6 | 16.9 | 15.4-18.5 | 81.5 | 79.2-83.7 | 18.5 | 16.3-20.8 |
| Peru | 57,348 | 4.8 | 2.4-9.3 | 54.1 | 52.3-55.9 | 45.9 | 44.1-47.7 | 71.2 | 68.8-73.5 | 28.8 | 26.5-31.2 | 83.5 | 82.5-84.4 | 16.5 | 15.6-17.5 | 82.6 | 81.6-83.6 | 17.4 | 16.4-18.4 |
| Puerto Rico, U.S. | 29,290 | 0.5 | 0.3-0.9 | 51.2 | 48.5-53.9 | 48.8 | 46.1-51.5 | 63.4 | 59.2-67.5 | 36.6 | 32.5-40.8 | 87.7 | 86.0-89.2 | 12.3 | 10.8-14.0 | 87.3 | 85.7-88.8 | 12.7 | 11.2-14.3 |
| Uruguay | 19,534 | 0.6 | 0.3-1.2 | 39.3 | 38.2-40.4 | 60.7 | 59.6-61.8 | 52.2 | 47.8-56.5 | 47.8 | 43.5-52.2 | 86.6 | 85.1-88.0 | 13.4 | 12.0-14.9 | 83.2 | 76.7-88.3 | 16.8 | 11.7-23.3 |
| Venezuela | 18,050 | 4.3 | 2.2-8.1 | 44.4 | 42.0-46.8 | 55.6 | 53.2-58.0 | 57.9 | 54.6-61.1 | 42.1 | 38.9-45.4 | 84.2 | 83.1-85.2 | 15.8 | 14.8-16.9 | 85.5 | 84.4-86.5 | 14.5 | 13.5-15.6 |

95%CI: 95% confidence intervals.

| Table S2. Proportion of compliance with secondary outcomes according to the countries of the study sample. | | | | | | | | |
| --- | --- | --- | --- | --- | --- | --- | --- | --- |
|  | **Isolation due to contact with a respiratory symptomatic** | | | | **Isolation due to symptoms** | | | |
|  | Yes | | No | | Yes | | No | |
| Countries | % | IC95% | % | IC95% | % | IC95% | % | IC95% |
| Argentina | 18.1 | 14.4-22.6 | 81.9 | 77.4-85.6 | 21.4 | 19.5-23.6 | 78.6 | 76.4-80.5 |
| Bolivia | 16.5 | 13.2-20.5 | 83.5 | 79.5-86.8 | 21.9 | 20.2-23.7 | 78.1 | 76.3-79.8 |
| Brazil | 14.8 | 13.8-15.9 | 85.2 | 84.1-86.2 | 16.8 | 15.8-17.8 | 83.2 | 82.2-84.2 |
| Chile | 23.2 | 16.6-31.4 | 76.8 | 68.6-83.4 | 23.2 | 19.4-27.6 | 76.8 | 72.4-80.6 |
| Colombia | 18 | 15.8-20.5 | 82 | 79.5-84.2 | 22.3 | 20.7-24.1 | 77.7 | 75.9-79.3 |
| Costa Rica | 10.7 | 8.6-13.3 | 89.3 | 86.7-91.4 | 15.2 | 14.1-16.5 | 84.8 | 83.5-85.9 |
| Dominican Republic | 22.4 | 16.3-30.1 | 77.6 | 69.9-83.7 | 24.2 | 21.9-26.7 | 75.8 | 73.3-78.1 |
| Ecuador | 23.4 | 21.7-25.2 | 76.6 | 74.8-78.3 | 26.9 | 25.1-28.8 | 73.1 | 71.2-74.9 |
| El Salvador | 25.3 | 22.7-28.1 | 74.7 | 71.9-77.3 | 25.9 | 23.3-28.7 | 74.1 | 71.3-76.7 |
| Guatemala | 13.6 | 10.1-18.0 | 86.4 | 82.0-89.9 | 16.4 | 14.5-18.5 | 83.6 | 81.5-85.5 |
| Haiti | 1.5 | 0.2-13.1 | 98.5 | 86.9-99.8 | 10.9 | 7.4-15.7 | 89.1 | 84.3-92.6 |
| Honduras | 28.2 | 21.1-36.6 | 71.8 | 63.4-78.9 | 26.7 | 24.1-29.5 | 73.3 | 70.5-75.9 |
| Mexico | 15.3 | 14.3-16.2 | 84.7 | 83.8-85.7 | 16.7 | 16.1-17.4 | 83.3 | 82.6-83.9 |
| Nicaragua | 9.3 | 7.7-11.3 | 90.7 | 88.7-92.3 | 10 | 7.1-14.0 | 90 | 86.0-92.9 |
| Panama | 22.7 | 13.5-35.7 | 77.3 | 64.3-86.5 | 29 | 23.2-35.7 | 71 | 64.3-76.8 |
| Paraguay | 20.6 | 14.2-28.9 | 79.4 | 71.1-85.8 | 17.1 | 14.8-19.7 | 82.9 | 80.3-85.2 |
| Peru | 22.2 | 20.1-24.4 | 77.8 | 75.6-79.9 | 23.6 | 22.2-25.0 | 76.4 | 75.0-77.8 |
| Puerto Rico, U.S. | 17.1 | 13.6-21.3 | 82.9 | 78.7-86.4 | 19.3 | 16.7-22.3 | 80.7 | 77.7-83.3 |
| Uruguay | 8.4 | 6.0-11.7 | 91.6 | 88.3-94.0 | 15.2 | 13.5-17.2 | 84.8 | 82.8-86.5 |
| Venezuela | 13 | 7.6-21.3 | 87 | 78.7-92.4 | 20.7 | 18.0-23.7 | 79.3 | 76.3-82.0 |

95%CI: 95% confidence intervals.

| Table S3. Bivariate analysis of the characteristics of the population according to the secondary outcomes in the study sample. | | | | | | | | | | |
| --- | --- | --- | --- | --- | --- | --- | --- | --- | --- | --- |
|  | **Isolation due to contact with a respiratory symptomatic** | | | | | **Isolation due to symptoms** | | | | |
|  | Yes | | No | |  | Yes | | No | |  |
| Characteristics | Weighted proportion according to each category | | Weighted proportion according to each category | | p value | Weighted proportion according to each category | | Weighted proportion according to each category | | p value |
|  | % | 95%CI | % | 95%CI |  | % | 95%CI | % | 95%CI |  |
| Gender |  |  |  |  | <0.001 |  |  |  |  | <0.001 |
| Male | 13.3 | 12.4-14.2 | 86.7 | 85.8-87.6 |  | 15.4 | 14.7-16.1 | 84.6 | 83.9-85.3 |  |
| Female | 18.8 | 17.6-20.2 | 81.1 | 79.8-82.4 |  | 21.1 | 20.0-22.3 | 78.9 | 77.7-80.0 |  |
| No binary | 11.6 | 8.9-14.9 | 88.4 | 85.1-91.1 |  | 19.6 | 18.0-21.2 | 80.4 | 78.8-82.0 |  |
| Age (years) |  |  |  |  | <0.001 |  |  |  |  | 0.010 |
| 18-24 | 19.8 | 18.1-21.6 | 80.2 | 78.4-81.9 |  | 22.9 | 21.9-23.9 | 77.1 | 76.1-78.1 |  |
| 25-34 | 15.2 | 14.3-16.2 | 84.8 | 83.8-85.7 |  | 16.9 | 16.2-17.6 | 83.1 | 82.4-83.8 |  |
| 35-44 | 13.7 | 12.4-15.3 | 86.3 | 84.7-87.6 |  | 15.3 | 14.2-16.4 | 84.7 | 83.6-85.8 |  |
| 45-54 | 14 | 12.8-15.3 | 86 | 84.7-87.2 |  | 16.4 | 15.1-17.8 | 83.6 | 82.2-84.9 |  |
| 55-64 | 18.4 | 15.9-21.2 | 81.6 | 78.8-84.1 |  | 21 | 19.4-22.7 | 79 | 77.3-80.6 |  |
| 65-74 | 23.1 | 17.9-29.3 | 76.9 | 70.7-82.1 |  | 26.6 | 24.3-29.2 | 73.4 | 70.8-75.7 |  |
| 75 years or older | 16.9 | 7.9-32.4 | 83.1 | 67.6-92.1 |  | 33.7 | 26.4-41.9 | 66.3 | 58.1-73.6 |  |
| Area of residence |  |  |  |  | 0.297 |  |  |  |  | 0.009 |
| City | 16.3 | 15.2-17.4 | 83.7 | 82.6-84.8 |  | 18.7 | 17.7-19.8 | 81.3 | 80.2-82.3 |  |
| Town | 15.2 | 13.9-16.6 | 84.8 | 83.4-86.1 |  | 18.4 | 17.4-19.3 | 81.6 | 80.7-82.6 |  |
| Village or rural area | 15.2 | 13.4-17.3 | 84.8 | 82.7-86.6 |  | 20.8 | 19.6-22.0 | 79.2 | 19.6-22.0 |  |
| Anxiety symptomatology |  |  |  |  | 0.337 |  |  |  |  | 0.076 |
| Yes | 16.3 | 15.2-17.5 | 83.7 | 82.5-84.8 |  | 18.9 | 17.8-20.0 | 81.1 | 80.0-82.2 |  |
| No | 15.8 | 14.9-16.8 | 84.2 | 83.2-85.1 |  | 18.7 | 17.9-19.5 | 81.3 | 80.5-82.1 |  |
| Depressive symptomatology | |  |  |  | 0.001 |  |  |  |  | 0.883 |
| Yes | 16.8 | 15.7-17.9 | 83.2 | 82.1-84.3 |  | 19.2 | 18.3-20.2 | 80.8 | 79.8-81.7 |  |
| No | 15.1 | 14.1-16.2 | 84.9 | 83.8-85.9 |  | 17.9 | 17.0-18.8 | 82.1 | 81.2-83.0 |  |
| Level of CMS applied |  |  |  |  | <0.001 |  |  |  |  | <0.001 |
| Low | 14.6 | 13.6-15.8 | 85.4 | 84.2-86.4 |  | 16.4 | 15.7-17.2 | 83.6 | 82.8-84.3 |  |
| Intermediate | 14.8 | 13.8-15.8 | 85.2 | 84.2-86.2 |  | 16.7 | 15.8-17.7 | 83.3 | 82.3-84.2 |  |
| High | 20.7 | 19.2-22.2 | 79.3 | 77.8-80.8 |  | 23.0 | 22.1-23.9 | 77.0 | 76.1-77.9 |  |
| COVID-19 symptomatology | |  |  |  | 0.036 |  |  |  |  |  |
| No | 15.4 | 14.4-16.4 | 84.6 | 83.6-85.6 |  |  |  |  |  |  |
| Yes | 16.7 | 15.5-18.0 | 83.3 | 82.0-84.5 |  |  |  |  |  |  |

95%CI: 95% confidence intervals.

| Table S4. Sensitivity analysis for missing data according in the study sample. | | | | | |
| --- | --- | --- | --- | --- | --- |
|  | Non-missing’s | | Missing’s | |  |
| Characteristics | Weighted proportion according to each category | | Weighted proportion according to each category | | p value |
|  | % | 95%CI | % | 95%CI |  |
| Gender |  |  |  |  | <0.001 |
| Male | 95.9 | 95.6-96.2 | 4.1 | 3.8-4.4 |  |
| Female | 94.9 | 94.5-95.3 | 5.1 | 4.7-5.5 |  |
| No binary | 92.9 | 91.3-94.2 | 7.1 | 5.8-8.7 |  |
| Age (years) |  |  |  |  | <0.001 |
| 18-24 | 96.0 | 95.8-96.3 | 4.0 | 3.7-4.2 |  |
| 25-34 | 95.7 | 95.4-96.0 | 4.3 | 4.0-4.6 |  |
| 35-44 | 94.8 | 94.5-95.2 | 5.2 | 4.8-5.5 |  |
| 45-54 | 94.9 | 94.3-95.4 | 5.1 | 4.6-5.7 |  |
| 55-64 | 95.4 | 95.0-95.8 | 4.6 | 4.2-5.0 |  |
| 65-74 | 95.2 | 94.7-95.6 | 4.8 | 4.4-5.3 |  |
| 75 years or older | 92.6 | 90.4-94.4 | 7.4 | 5.6-9.6 |  |
| Area of residence | |  |  |  | <0.001 |
| City | 95.7 | 95.4-96.1 | 4.3 | 3.9-4.6 |  |
| Town | 95.8 | 95.5-96.1 | 4.2 | 3.9-4.5 |  |
| Village or rural area | 94.8 | 94.3-95.2 | 5.2 | 4.8-5.7 |  |
| Anxiety symptomatology | |  |  |  | <0.001 |
| Yes | 95.3 | 94.9-95.7 | 4.7 | 4.3-5.1 |  |
| No | 96.8 | 96.5-97.0 | 3.2 | 3.0-3.5 |  |
| Depressive symptomatology | | |  |  | <0.001 |
| Yes | 95.3 | 94.9-95.7 | 4.7 | 4.3-5.1 |  |
| No | 96.6 | 96.3-96.8 | 3.4 | 3.2-3.7 |  |
| Level of CMS applied |  |  |  |  | <0.001 |
| Low | 94.5 | 94.1-94.8 | 5.5 | 5.2-5.9 |  |
| Intermediate | 93.0 | 92.4-93.6 | 7.0 | 6.4-7.6 |  |
| High | 92.8 | 92.3-93.2 | 7.2 | 6.8-7.7 |  |
| COVID-19 symptomatology |  |  |  |  | 0.439 |
| No | 98.4 | 98.3-98.5 | 1.6 | 1.5-1.6 |  |
| Yes | 98.5 | 98.4-98.5 | 1.5 | 1.5-1.6 |  |
| Physical distancing |  |  |  |  | <0.001 |
| No | 95.3 | 94.9-95.6 | 4.7 | 4.4-5.1 |  |
| Yes | 95.8 | 95.4-96.1 | 4.2 | 3.9-4.6 |  |
| Hand washing |  |  |  |  | 0.433 |
| No | 95.4 | 95.1-95.6 | 4.6 | 4.4-4.9 |  |
| Yes | 95.5 | 95.1-95.8 | 4.5 | 4.2-4.9 |  |
| Mask or face covering use |  |  |  |  | 0.903 |
| No | 95.5 | 95.1-95.9 | 4.5 | 4.1-4.9 |  |
| Yes | 95.5 | 95.2-95.8 | 4.5 | 4.2-4.8 |  |
| Compliance with the principal mitigation measures |  |  |  |  | <0.001 |
| No | 94.8 | 94.4-95.1 | 5.2 | 4.9-5.6 |  |
| Yes | 95.3 | 95.1-95.7 | 4.7 | 4.3-5.1 |  |

95%CI: 95% confidence intervals.

**Supplementary file: Questionnaire**

1. You understand the above and consent to take part in this survey run by the University of Maryland and Johns Hopkins University.
   1. Yes
   2. No
2. Do you consent with sharing your data with these academic institutions?
   1. Yes
   2. No
3. You must be 18 years or older to take this survey. Are you 18 years or older?
   1. Yes
   2. No
4. What is the country or region where you are currently staying?
   1. See country region response map file.
5. What is your gender?
   1. Male
   2. Female
   3. Other
6. What is your age?
   1. 18-24 years
   2. 25-34 years
   3. 35-44 years
   4. 45-54 years
   5. 55-64 years
   6. 65-74 years
   7. 75 years or older
7. Which of these best describes the area where you are currently staying?
   1. City
   2. Town
   3. Village or rural area
8. In the last 24 hours, have you had direct contact with anyone who is not staying with you? Direct contact means spending longer than one minute within two meters of someone or touching, including shaking hands, hugging, or kissing.
   1. Yes
   2. No
9. In the past 7 days, how often did you wear a mask when in public?
   1. All of the time
   2. Most of the time
   3. Some of the time
   4. A little of the time
   5. None of the time
   6. I have not been in public during the past 7 days
10. In the last 7 days, how often did you wash your hands with soap after being in public?
    1. All of the time
    2. Most of the time
    3. Some of the time
    4. A little of the time
    5. None of the time
    6. I have not been in public during the past 7 days
11. During the past 7 days, how often did you feel so nervous that nothing could calm you down?
    1. All of the time
    2. Most of the time
    3. Some of the time
    4. A little of the time
    5. None of the time
12. During the past 7 days, how often did you feel so depressed that nothing could cheer you up?
    1. All of the time
    2. Most of the time
    3. Some of the time
    4. A little of the time
    5. None of the time
13. In the past 24 hours, have you personally experienced any of the following symptoms?
    1. Fever
    2. Cough
    3. Difficulty breathing
    4. Fatigue
    5. Stuffy or runny nose
    6. Aches or muscle pain
    7. Sore throat
    8. Chest pain
    9. Nausea
    10. Loss of smell or taste
    11. Eye pain
    12. Headache
14. Do you personally know anyone in your local community who is sick with a fever and at least one other symptom?
    1. Yes
    2. No
15. Have you spent time with any of these people in the last 7 days?
    1. Yes
    2. No
16. In the last 24 hours, have you done any of the following? Gone to work outside the place where you are currently staying
    1. Yes
    2. No
17. In the last 24 hours, have you done any of the following? Gone to a market, grocery store, or pharmacy
    1. Yes
    2. No
18. In the last 24 hours, have you done any of the following? Gone to a restaurant, cafe, or shopping center
    1. Yes
    2. No
19. In the last 24 hours, have you done any of the following? Spent time with someone who isn't currently staying with you
    1. Yes
    2. No
20. In the last 24 hours, have you done any of the following? Attended a public event with more than 10 people
    1. Yes
    2. No
21. In the last 24 hours, have you done any of the following? Used public transit
    1. Yes
    2. No
